# Early and late mortality in vertebral osteomyelitis: who dies within the first year after diagnosis

**DOI:** 10.1007/s15010-025-02541-9

**Published:** 2025-05-09

**Authors:** Nikolaus Kernich, Arian Abi-Chokami, Norma Jung, Dorothee Jochimsen, Krishnan Sircar, Ada Marie Hoffmann, Stefan Meuser, Peer Eysel, Carolyn Weber, Juan Manuel Vinas-Rios, Ayla Yagdiran, on behalf of the Interdisciplinary Studygroup of Spondylodiscitis – Cologne (IST-SPONDYL), Norma Jung, Ayla Yagdiran, Nikolaus Kernich, Krishnan Sircar, Julia Fischer, Dorothee Jochimsen, Carolyn Weber, Charlotte Meyer-Schwickerath

**Affiliations:** 1 Department of Orthopedics and Trauma Surgery, Faculty of Medicine and University Hospital of Cologne, University of Cologne, Cologne, Germany; 2 Division of Infectious Diseases, Department I of Internal Medicine, Faculty of Medicine and University Hospital of Cologne, University of Cologne, Cologne, Germany; 3 Institute for Medical Microbiology, Immunology and Hygiene, Faculty of Medicine and University Hospital of Cologne, Cologne, Germany; 4 Department of Hand Surgery, Helios Bonn/ Rhein Sieg, Bonn, Germany; 5 Department of Cardiothoracic Surgery, Faculty of Medicine and University Hospital of Cologne, University of Cologne, Cologne, Germany

**Keywords:** Vertebral osteomyelitis, Spondylodiscitis, Surgery, Early mortality, Late mortality, Risk factors

## Abstract

**Objective:**

Vertebral osteomyelitis (VO) is a severe clinical entity associated with high mortality rates, especially within the first year after diagnosis. The aim of this single-center prospective cohort study was to identify and compare predictive factors influencing early and late mortality in patients with conservatively and surgically treated VO.

**Methods:**

We conducted a single-center prospective cohort study including patients treated for VO between 2008 and 2020 in a tertiary center in Germany to determine early (death within 30 days after diagnosis) and late mortality (death between day 31 and 365 after diagnosis). Additionally, multivariable analyses were performed to analyze predictive risk factors for early and late mortality.

**Results:**

A total of 323 patients were included. 19% died within the first year after diagnosis. Early mortality occurred in 5% and late mortality in 14% of cases. Multivariable analysis revealed chronic kidney disease (CKD) (OR: 13.2, 95% CI 5.7–30.3; p < 0.001) and MSSA (OR: 4.0, 95% CI 1.4–11.1; p = 0.008) as independent risk factors for early mortality, whereas ASA score > 2 (HR: 5.2, 95% CI 2.6–10.6; p < 0.001), age > 70 years (HR: 2.4, 95% CI 1.6–3.7; p < 0.001), CKD (HR: 1.9, 95% CI 1.3–3.0; p = 0.003) and bacteremia (HR: 1.8, 95% CI 1.2–2.7; p = 0.002) were identified as independent risk factors for late mortality.

**Conclusion:**

One out of five VO patients dies within the first year after diagnosis. Risk factors for mortality within the first year include CKD and bacteremia. As a consequence in particular those patients should be closely monitored within the first year after.

## Introduction

### Background/rationale

Vertebral osteomyelitis (VO) is a serious disease associated with a significant morbidity and mortality [1–3]. The increasing incidence is directly related to demographic changes and multimorbidity in the elderly [4], which causes an increase in spinal disease and spinal interventions [5]. 

The likelihood of suffering VO increases significantly with the presence of certain risk factors. Patient-associated factors include biographical and modifiable risk factors as well as favoring diseases. One of the most important biographic risk factors is age. The average age at onset of disease ranges from 50.4 to 69.2 years and most infections occur in the 5th to 7th decade of life [4, 6–8]. The incidence of VO significantly increases after the age of 60 years [4]. Despite advances in the diagnosis and treatment of VO, mortality remains high at up to 24% within the first year after diagnosis [2, 6, 9, 10]. However, studies do not differentiate between in-hospital-mortality and mortality after discharge. We previously reported the mean length of stay of VO patients to be approximately 30 days [10]. We therefore defined early mortality as death within 30 days of diagnosis and late mortality as death from 31 to 365 days after diagnosis.

The aim of this work was (i) to determine the rate of early and late mortality in VO, (ii) to survey differences in patient characteristics of patients who died early or late after treatment of VO, and (iii) to identify independent risk factors for early or late mortality. Insight into this could improve and individualize monitoring and risk management of VO patients and therefore reduce mortality.

## Materials and methods

### Study design and data collection

This study is based on prospectively collected data from the European Spine Registry, formerly “Spine Tango” and the German Spine Registry of the German Spine Society (= DWG), collected between 2008 and 2020. Data were collected in a tertiary referral hospital in Germany with a specialization for the treatment of VO. Adult patients with a diagnosis of VO were included. The diagnosis was discussed and confirmed on an interdisciplinary basis by both infectious and orthopedic specialists. Data collection and case verification was carried out as already described by our working group [11, 12]. Patients were followed up 3 and 12 months after discharge, including an X-Ray and an annual telephone interview. CKD was defined and classified according to the KDIGO classification (Kidney Disease—Improving Global Outcomes) and KDIGO 2012 guidelines. CKD was present if the glomerular filtration rate was reduced to below 60 ml/min/1.73m^2^ over a period of at least 3 months. In this study, we did not distinguish between the stages of CKD or the need for dialysis, as the number of cases would then have been too small to achieve sufficient statistical power.

### Case verification

The plausibility and relevance of the detected pathogens were verified by an infectiologist. While low virulent organisms (e.g. coagulase negative staphylococci or propionibacteria) required a pathogen detection in at least two samples to confirm the infection, a single detection was sufficient for virulent organisms such as *S. aureus* and gram-negative bacteria. Anti-infective therapy adapted to the pathogen was administered after identification of the pathogen. An exception was the presence of severe sepsis. In this case, empirical anti-infective therapy was started preoperatively after blood-cultures were obtained. By default, a two-week intravenous antibiotic therapy was given postoperatively. Subsequently, the therapy was administered orally and continued for six to twelve weeks, depending on the severity of the disease, response to therapy, and risk factors (e.g., infections associated with foreign material). Exceptions included complicated courses, such as concomitant IE, *S. aureus* bloodstream infection, and disseminated infections, as well as treatment of specific microorganisms. The former required prolonged intravenous therapy and the latter required a therapeutic regimen adapted to the pathogen according to guidelines. Therapeutic success was determined by clinical and laboratory parameters. Pain relief and significant reduction of inflammatory values, especially CRP, indicated a response to therapy. If parameters remained unchanged, a control MRI was performed.

### Institutional protocol for diagnosis

In our institution, MRI is the gold standard for confirming the diagnosis of VO. Since our hospital is a maximum care provider, most patients are already referred with the appropriate diagnosis/imaging. If MRI is not yet available and there is clinical suspicion of VO, we systematically arrange for all patients to have an MRI performed in a timely manner. In addition to routine blood cultures before, during and after therapy, every patient with proven spondylodiscitis will receive an echocardiography to rule out concomitant infective endocarditis.

### Surgical procedure

The decision for surgical treatment was based on well-defined criteria. Unless bony destruction, spinal instability, or empyema were evident, a conservative therapeutic approach was chosen. The main reasons for surgical intervention were neurological deficits. However, progressive spinal deformities, (painful) spinal instability, intraspinal empyema, or unsuccessful conservative treatments also indicated surgery. All patients with progressive neurologic deficits were immediately referred for surgical treatment. Surgical treatment was performed according to the vertebral destruction. The indication for single- or two-stage surgical treatment was dependent on the extent of the bony destruction: If only an abscess or even an empyema was present, intraspinal debridement was performed. In cases of bony destruction and/or instability additional intercorporal fusion was carried out. If a reconstruction of the ventral column was necessary but not possible from the dorsal approach due to the extent of the bony destruction, a two- staged treatment was chosen: debridement and instrumentation in the first surgery and corporectomy/spondylodesis with implantation of iliac crest grafts, intervertebral cages or vertebral body replacement in the second surgery (ventral approach).

### Antibiotic treatment

Except for patients with severe sepsis, no empirical anti-infective therapy was recommended preoperatively to identify the specimen. Whenever possible, antimicrobial therapy of VO was directed against the identified specimen. In culture negative cases, empirical therapy was administered with our local standard regimen (ceftriaxone and flucloxacillin) as MRSA rates are low in Germany. In general, postoperative antibiotic treatment was administered intravenously for 14 days followed by a highly bioavailable oral antimicrobial therapy oralization with a duration of 6 to 12 weeks in total depending on the severity of diseases deferred response or risk factors (e.g., foreign material-associated infection). A longer intravenously therapy was applied when indicated (e.g., concomitant endocarditis; Staphylococcus aureus bacteremia; disseminated infectious complications). Therapy for specific microorganisms was applied according to the guidelines (e.g., Mycobacterium spp., Candida spp., Brucella spp.). The response to therapy was controlled clinically (pain reduction) and by laboratory inflammation marker (significant CRP drop). All patients received an infectious disease consultation or were discussed in our osteomyelitis board and received anti-infective therapy according to interdisciplinary consensus.

### Statistical methods

Continuous variables are described as mean (± standard deviation) or median (interquartile range), according to the normality of their distribution. Variables were compared using unpaired t-test or Mann–Whitney-U-test as appropriate. Discrete variables were tested with Pearson’s chi-square test or, when validity conditions were not satisfied, with Fisher’s exact test. Discrete Variables are reported as absolute number (percentage). Missing data were not imputed and assumed to be missing at random. only patients with a complete 1-year follow-up were included in the analysis. We have chosen 30-day mortality as an “early mortality” measure because it allows the assessment of acute risks and the effectiveness of treatment approaches in the early phase. Compared to in-hospital mortality, it offers better comparability because deaths within a specified period are standardized independently of the length of hospital stay. In-hospital mortality, on the other hand, can be distorted by differences in discharge practices (earlier transfer to rehabilitation facilities or nursing homes). The one-year mortality as “late mortality” rate provides information about the long-term effects of the disease and its treatments. It helps to assess the sustainability of treatment outcomes and to understand how the disease affects the patient’s quality of life and general state of health over a longer period of time. Potential risk factors for early mortality (day 1–30) were assessed using logistic regression, potential risk factors for late mortality (day 31–365) using Cox regression. After univariate analysis, all variables with a p-value less than 0.05 were entered into the multivariable model using forward selection (likelihood ratio, p_in_ = 0.05). Results are presented as odds ratio for early mortality or hazard ratio for late mortality with corresponding 95% confidence intervals and p-values. For the independent predictors emerging from the MVA, Kaplan–Meier curves were used to graphically display the survival times. The survival time in days of the various subgroups was displayed as horizontal lines and censored cases were displayed as vertical lines. The log-rank test was used to show differences in long-term mortality in patients with/without bacteremia, chronic kidney disease, age > 70 years, and American Society of Anesthesiologists (ASA) score > 2. All reported p-values are 2-sided. Statistical analyses were performed using SPSS Statistics Version 28.

### Ethics

Ethical approval was given by our local ethics committee (Ruling No. 09-182). Written informed consent for inclusion in the registry was obtained from all patients.

## Results

### Patients characteristics and manifestation of VO

**Fig. 1:**
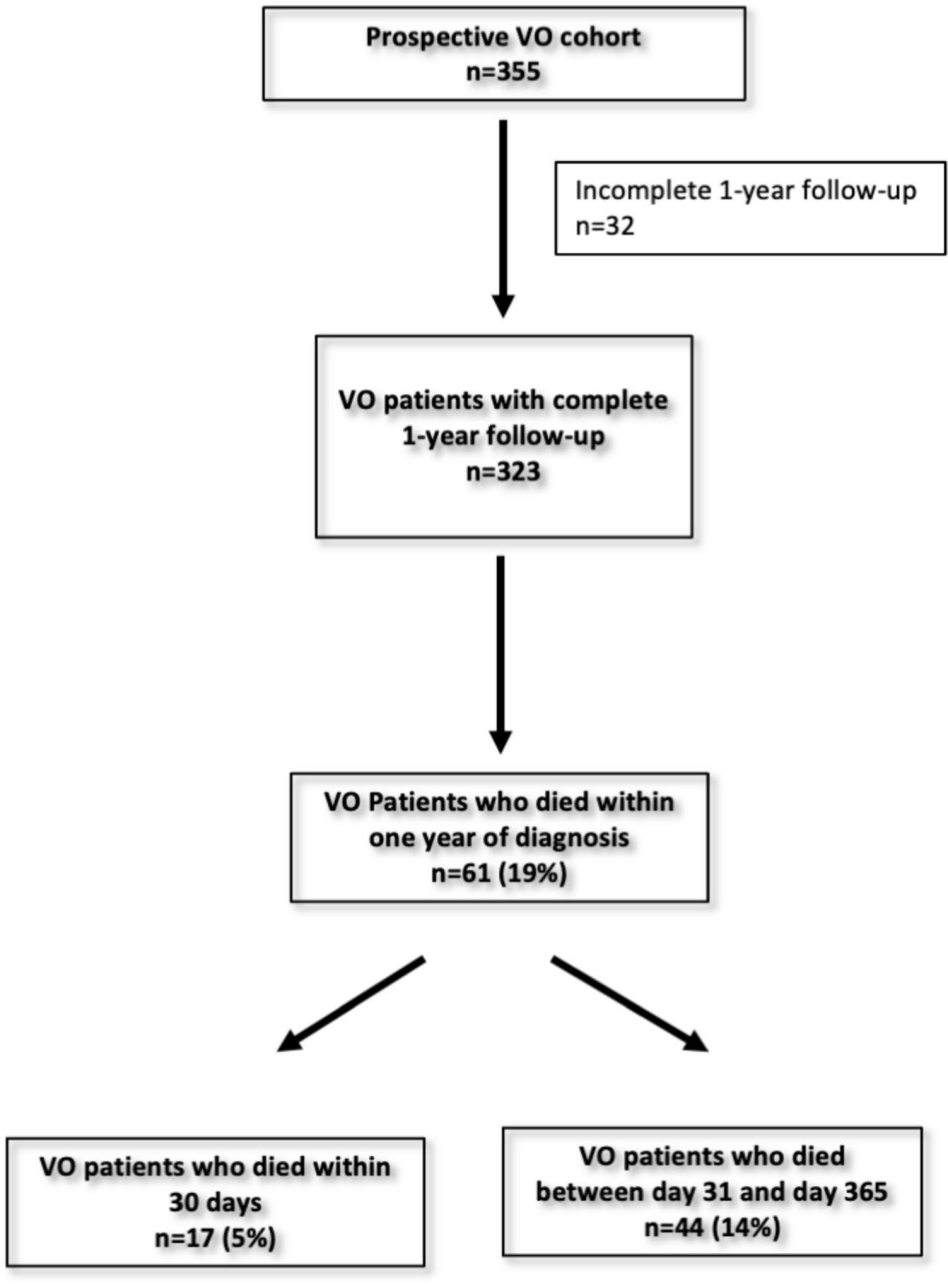
Flowchart of VO patients who died within the first year after diagnosis

Between 2008 and 2020, 355 patients with a confirmed diagnosis of VO were treated at our institution. Of these patients, mortality information was available for 323 within the first year (Fig. 1).

Demographics and clinical characteristics are shown in Table 1. Of all patients included, 64% were male, the median age was 69 [60–76] years. Compared to the overall collective, patients who died within the first 30 days had more often an ASA score of 4 (31% vs. 10%; p = 0.018), chronic kidney disease (CKD) (53% vs. 15%; p < 0.001) and more than two comorbidities (59% vs. 36%; p = 0.046). Patients who died between day 31 and day 365 were older (median 74 years vs. median 69 years; p < 0.001), had more often an ASA score of 4 (26% vs. 10%; p = 0.002), a higher rate of heart failure (30% vs. 16%; p = 0.011), CKD (32% vs. 15%; p < 0.001) and more than two comorbidities (61% vs. 36%; p < 0.001).

**Table 1:** Characteristics of VO patients with early (between diagnosis and day 30) and late (between day 31 and day 365) mortality

	All VO patients (n = 323)	Patients who died within 30 days (n = 17/323)	p-value	Patients who died between day 31 and 365 (n = 44/323)	p-value*
Age (years)					
Median [interquartile range]	69 [60; 76]	67 [78; 82]	**0.010**	74 [66; 79]	** < 0.001**
Age > 70	152/323 (47)	11/17 (65)	0.134	29/44 (66)	**0.007**
Male n (%)	207/323 (64)	11/17 (65)	0.956	24/44 (55)	0.156
BMI					
Median [interquartile range]	26 [23; 30]	24 [20; 31]	0.453	26 [22; 31]	0.664
BMI > 25	164/288 (57)	7/14 (50)	0.591	22/38 (58)	0.899
ASA score n (%)					
1	12/308 (4)	0/16 (0)	0.253	0/43 (0)	0.055
2	91/308 (30)	1/16 (6)	**0.017**	3/43 (7)	** < 0.001**
3	173/308 (56)	10/16 (63)	0.600	29/43 (67)	0.108
4	32/308 (10)	5/16 (31)	**0.018**	11/43 (26)	**0.002**
Underlying comorbidities					
Diabetes n (%)	77/323 (24)	4/17 (24)	0.975	16/44 (36)	**0.036**
Malignancy n (%)	68/323 (21)	6/17 (35)	0.164	11/44 (25)	0.490
Rheumatic disease n (%)	22/323 (7)	1/17 (6)	0.873	4/44 (9)	0.534
Heart failure n (%)	53/323 (16)	5/17 (29)	0.169	13/44 (30)	**0.011**
COPD n (%)	33/323 (10)	2/17 (12)	0.832	10/44 (23)	**0.008**
Chronic kidney disease n (%)	49/323 (15)	9/17 (53)	** < 0.001**	14/44 (32)	** < 0.001**
Alcohol disorder n (%)	24/323 (7)	0/17 (0)	0.100	5/44 (11)	0.312
Drug disorder n (%)	11/323 (3)	0/17 (0)	0.271	0/44 (0)	0.070
Number of comorbidities					
0 n (%)	110/323 (34)	2/17 (12)	**0.046**	5/44 (11)	** < 0.001**
1 n (%)	96/323(30)	5/17 (29)	0.977	12/44 (27)	0.702
≥ 2 n (%)	117/323 (36)	10/17 (59)	**0.046**	27/44 (61)	** < 0.001**
Bacteremia n (%)	113/322 (35)	11/17 (65)	**0.009**	26/44 (59)	** < 0.001**
MSSA	53/322 (17)	8/17 (47)	**0.002**	12/44 (27)	**0.037**
MRSA	8/322 (3)	1/17 (6)	0.425	2/44 (5)	0.387
CoNS	24/322 (8)	1/17 (6)	0.793	5/44 (11)	0.315
Gramnegative species	5/322 (2)	0/17 (0)	0.460	3/44 (7)	0.173
* Enterococcus* species	10/322 (3)	0/17 (0)	0.294	2/44 (5)	0.142
* Proprionibacterium* species	1/322 (0)	0/17 (0)	0.742	0/44 (0)	0.587
Anaerobes	2/322 (1)	0/17 (0)	0.641	0/44 (0)	0.443
* Streptococcus* species	9/322 (3)	1/17 (6)	0.484	2/44 (5)	0.480
Spinal culture n (%)	236/323 (73)	15/17 (88)	0.117	33/44 (75)	0.756
* S. aureus*	95/231 (41)	11/15 (73)	**0.009**	17/31 (55)	0.095
MSSA	86/231 (37)	10/15 (67)	**0.015**	15/31(49)	0.167
MRSA	9/231 (4)	1/15 (7)	0.599	2/31 (7)	0.121
CoNS	51/231 (22)	2/15 (13)	0.373	5/31 (16)	0.391
Gramnegative species	20/231 (9)	1/15 (7)	0.769	2/31 (7)	0.626
* Enterococcus* species	18/231 (8)	0/15 (0)	0.112	3/31 (10)	0.683
Mycobacteria	8/231 (4)	0/15 (0)	0.296	1/31 (3)	0.937
* Proprionibacterium* species	9/231 (4)	0/15 (0)	0.267	0/31 (0)	0.104
Anaerobes	9/231 (4)	0/15 (0)	0.267	0/31 (0)	0.104
* Streptococcus* species	17/231 (7)	1/15 (7)	0.914	3/31 (10)	0.609
* Candida* species	1/231 (1)	0/15 (0)	0.524	0/31 (0)	0.351
* Corynebacterium s*pecies	1/231 (0.4)	0/15 (0)	0.714	0/31 (0)	0.591
ID Consultation n (%)	269/323 (83)	13/17 (77)	0.459	37/44 (84)	0.876
Manifestations n (%)					
Endocarditis	17/323 (5)	0/17 (0)	0.169	7/44 (16)	**0.004**
Psoas abscess	70/323 (22)	6/17 (35)	0.185	16/44 (36)	**0.011**
Empyema	108/323 (33)	7/17 (41)	0.487	13/44 (30)	0.556
Spinal level					
Cervical n (%)	10/322 (3)	0/17 (0)	0.294	1/44 (2)	0.721
Thoracic n (%)	90/322 (28)	6/17 (35)	0.498	17/44 (39)	0.089
Lumbar n (%)	207/322 (64)	10/17 (59)	0.629	22/44 (50)	**0.033**
Multilevel n (%)	25/322 (5)	1/17 (6)	0.812	4/44 (9)	0.172
Segments affected					
1 n (%)	253/323 (78)	14/17 (82)	0.672	35/44 (80)	0.833
> 1 n (%)	70/323 (22)	3/17 (18)	0.672	9/44 (20)	0.833
Prior infiltration	36/321 (11)	0/17 (0)	0.132	1/44 (2)	**0.018**
Prior operation	66/321 (21)	0/17 (0)	**0.005**	6/44 (14)	0.221
Foreign material placed	15/321 (5)	0/17 (0)	0.195	2/44 (5)	0.990
Neurological deficit (Frankel) n (%)					
A	4/323 (1)	0/17 (0)	0.509	1/44 (2)	0.542
B	17/323 (5)	4/17 (24)	**0.008**	7/44 (16)	**0.004**
C	27/323 (8)	1/17 (6)	0.691	4/44 (9)	0.852
D	26/323 (8)	1/17 (6)	0.725	2/44 (5)	0.325
E	249/323 (77)	11/17 (65)	0.234	30/44 (68)	0.130
Vertebral destruction (Eysel/Peters) n (%)					
1	28/318 (9)	2/16 (13)	0.611	0/43 (0)	**0.003**
2	167/318 (53)	8/16 (50)	0.836	17/43 (40)	0.067
3	123/318 (39)	6/16 (37)	0.921	26/43 (60)	**0.002**
Surgical treatment n (%)	384/323 (88)	16/17 (94)	0.379	35/44 (80)	0.066
Days until surgery	4 [2.0; 8.0]	5 [2; 20]	0.233	4 [2; 9]	0.478
Median [interquartile range]					
Single-stage spinal surgery n (%)	132/279 (47)	12/16 (75)	**0.022**	14/34 (41)	0.444
Chip	100/225 (44)	1/8 (13)	**0.047**	8/28 (29)	0.071
Cage	117/225 (52)	7/8 (88)	**0.029**	19/28 (68)	0.073
Chip + cage	8/225 (4)	0/8 (0)	0.442	1/28 (4)	0.996
Outcome					
Recurrence n (%)	24/323 (7)	0/17 (0)	0.100	2/44 (5)	0.405
Mortality					
Early (between diagnosis and day 30)	17/323 (5)				
Late (between day 31 and 365)	44/323 (14)				

Bold values were considered significant with a p<0.05

* values were considered significant with a p<0.05

Data presented as percent (number) or median [IQR], respectively

*ASA* American Society of Anesthesiologists, *BMI* body mass index, *CoNS* coagulase negative staphylococcus, *COPD* chronic obstructive pulmonary disease, *ID* infectious disease, *IQR* interquartile range, *VO* vertebral osteomyelitis

Further details on the level of spinal involvement, concomitant empyema or psoas abscesses are shown in Table 1. In the overall collective, concomitant IE occurred in 5%. Of the patients who died early, none had concomitant IE, whereas 16% (p = 0.004) of patients who died late suffered from concomitant IE. 

**Fig. 2:**
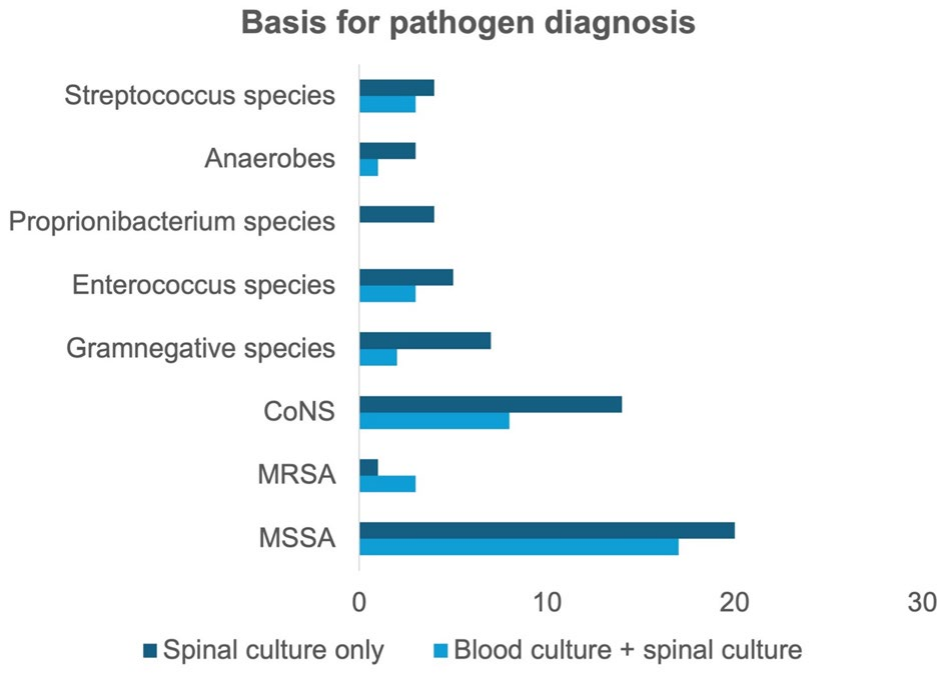
Basis for pathogen diagnosis in patients with VO

Pathogen detection in blood cultures was only achieved in 35% of patients. In all of these patients, pathogen detection was simultaneously achieved in intraoperative material. Overall, pathogen detection was achieved in 73% of patients from samples obtained intraoperatively. We have illustrated the distribution for the basis of pathogen detection in Fig. 2**.**

Compared to the overall population, patients who died early (65% vs. 35%; p = 0.009) or late (59% vs. 35%; p < 0.001) had a significantly higher rate of bacteremia. The most common pathogen observed was MSSA. MSSA was also most frequently detected in spinal cultures with 37%, followed by coagulase-negative Staphylococci (CoNS) (22%), gram-negative species (9%), Enterococci species (8%) and mycobacteria (3%). Patients who died within the first 30 days were significantly more likely to be infected with MSSA (67%) (p = 0.015) (Table 1).

Overall, 88% of the patients underwent surgery. Of these, 47% of surgical treatments were performed in a single-stage procedure. Among patients who died within the first 30 days, the rate of single-stage surgical treatment was significantly higher compared to the total group (75% vs. 47%, p = 0.022). 21% of the patients had spinal surgery before the episode of this VO, and 5% of the patients had foreign material inserted during the previous operation. In all patients in whom foreign material had previously been used, there was an association of VO with the foreign material introduced (Table 1).

### Prevalence of early or late mortality

The median follow-up time was 791 [565–1820] days with a maximum duration of up to 4614 days. A total of 61 patients died within the first year after diagnosis. This corresponds to a 1-year mortality rate of 19%. Of these, 5% died within the first 30 days after diagnosis, and another 14% died between days 31 and 365. (Table 1).

### Risk factors for early or late mortality

Multivariable analysis identified the presence of CKD (OR: 13.2, 95% CI 5.7–30.3; p < 0.001) as well as infection with *MSSA* (OR: 4.0, 95% CI 1.4–11.1; p = 0.008) as independent predictors of early mortality.

**Fig. 3:**
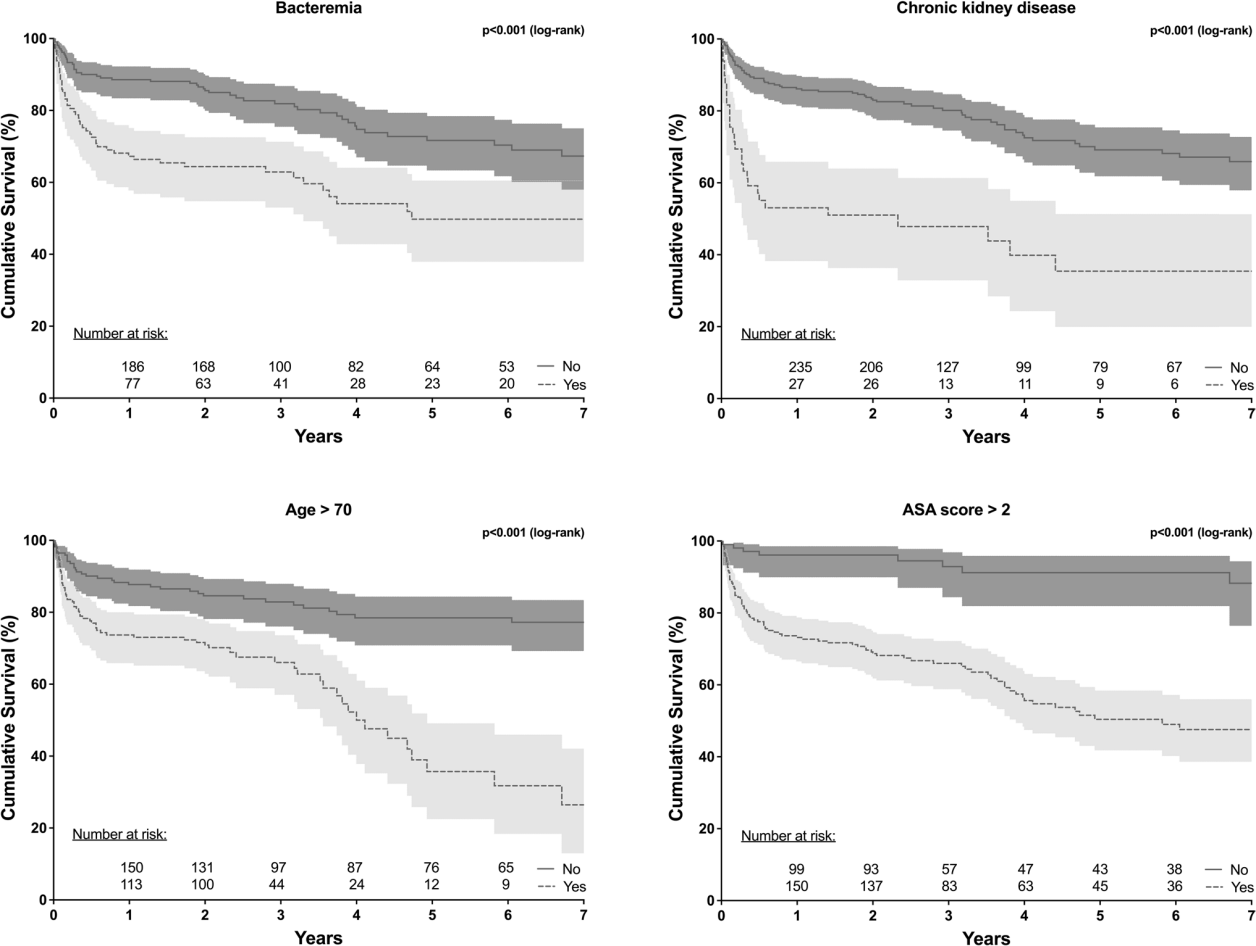
Kaplan Meier curves in the presence of independent predictors of increased mortality

The presence of CKD was also an independent predictor of late mortality (HR: 2.0, 95% CI 1.3–3.0; p = 0.003). Other predictors of late mortality were age over 70 years (HR: 2.4, 95% CI 1.6–3.7; p < 0.001), ASA score over 2 (HR: 5.2, 95%CI 2.6–10.6; p < 0.001) and bacteremia (HR: 1. 8, 95% CI 1.2–2.7; p = 0.002) (Table 2). The Kaplan Meier curves in Fig. 3 illustrate the limited survival in the presence of the above risk factors CKD, age > 70, ASA > 2 and bacteremia. The Kaplan Meier curves underline that mortality is most significant in the first year in the presence of risk factors (bacteremia, CKD, age > 70, ASA score > 2), as shown by the drop in the curves. After that, the curves run almost parallel.

**Table 2:** Multivariable analysis of independent predictors associated with early (between diagnosis and day 30) and late (between day 31 and day 365) mortality in VO patients

	Early mortality			Late mortality		
	OR	95% CI	P value	HR	95% CI	P value
Age > 70 years				2.417	1.575–3.708	** < 0.001**
ASA score > 2				5.237	2.588–10.597	** < 0.001**
Chronic kidney disease	13.188	5.741–30.291	** < 0.001**	1.950	1.254–3.032	**0.003**
Bacteremia				1.829	1.238–2.701	**0.002**
MSSA	4.013	1.447–11.126	**0.008**			

Bold values were considered significant with a p<0.05

*ASA* American Association of Anesthesiologists, *CI confidence interval, HR* hazard ratio, *OR* odds ratio, *VO* vertrebral osteomyelitis

The median survival for the VO total collective was 10.48 years. The presence of an ASA score > 2 reduced the median survival to 5.82 years. Bacteremia led to a median survival of 4.73 years, similar to age > 70 years, which showed a median survival of 4 years. The presence of CKD had the greatest impact on median survival, reducing it to 2.33 years.

## Discussion

We present a comprehensive analysis of VO patients from a prospective cohort focused on identifying and comparing predictors of early and late mortality. Our main findings were as follows: (i) Within the first 30 days, 5% of patients died, and another 14% died between day 31 and day 365. (ii) Patients who died within the first 30 days of diagnosis had a higher ASA score, a higher rate of CKD, were more likely to have bacteremia, and MSSA was detected more frequently as the causative agent. Patients who died between days 31 to 365 were older, more likely to have heart failure as well as CKD, and more likely to have bacteremia, concomitant IE, and psoas abscess. (iii) CKD and MSSA were identified as independent risk factors for early mortality. Our data suggest that late mortality did not seem to be clearly driven by recurrent VO, but other independent predictors such as ASA score > 2, age > 70 years, CKD, and bacteremia.

### Mortality

The mortality rates of this study are high compared with most other studies which range from 2 to 24%[2, 6, 13–15]. In contrast to our cohort, aforementioned studies had a high proportion of patients treated conservatively. It is noticeable that studies with a high ratio of surgical therapy show high mortality rates [10, 16]. Regarding the high rate of surgical interventions in our study, the mortality rate can be explained by the severity of the course of the disease and underlines the role of a tertiary care hospital: specialization of a spine center may facilitate the treatment of particularly complex cases that may not be adequately cared for in peripheral hospitals. The present analysis with 355 VO patients confirms the high 1-year mortality of 20% found by Kehrer et al. [2].

As only patients for whom a complete 1-year follow-up was available were included in this analysis, 9% of patients without a 1-year follow-up were excluded. Therefore the reported 1-year mortality may be slightly underestimated, since patients who are lost to follow-up often have a higher mortality. Nevertheless, since the 1-year mortality is in the range reported in the literature, it was important to us to focus on patients with complete follow-up to be able to provide a valid statement about the immediate postoperative and 1-year course after VO.

### Risk factors for early and late mortality

Previous studies have analyzed pre-existing conditions in VO and their influence on the course of the disease. Typically, VO occurs more often in multimorbid patients. Accordingly, the prevalences of diabetes, malignant tumor, heart failure, CKD, COPD, rheumatic disease, alcohol addiction, and drug disorder were higher in our cohort than in the normal population as also reported by Kehrer et al. [2]. While other studies revealed similar prevalence of comorbidities [2, 7, 17], the risk factors influencing the early and late mortality rate of VO are still only poorly understood.

#### Age and ASA score

In our study, age older than 70 years was found to be a predictive factor for death between day 31 and day 365. The significant impact of age on survival after VO diagnosis was also described in the study by Kehrer et al. [2]. They demonstrated an increased 1-year mortality starting at an age of 65 years. Furthermore, Akiyama et al. [18] and Loibl et al. [19] described a significant impact of an age over 60 years on in-hospital mortality. Issa et al. [13] also demonstrated significantly increasing probabilities of general death in VO patients older than 60 years in their epidemiological analysis. The rationale for the influence of age lies in increasing multimorbidity. The assumption is further strengthened by the associated ASA risk classification. Two thirds of patients were assigned to class 3 or worse with severe general or life-threatening diseases. In patients with VO, a higher ASA score is associated with a worse outcome [20]. This was confirmed in our study. We were able to show that an ASA score of > 2 was already an independent predictor of 1-year mortality.

#### Staphylococcus aureus

We were able to show that MSSA is a risk factor for early mortality and increases the probability of dying in the first month after diagnosis by fourfold. A reason for this may be the virulence factors of *S. aureus,* which lead to increased pathogenicity, confer the ability to form abscesses and are associated with more infection-related complications.

In 2014 Aagard et al. analyzed long-term mortality of VO patients surviving the first year after diagnosis and were able to demonstrate that VO caused by *S. aureus* leads to an increased mortality in bacteremic patients [9]. However, in our study we could not demonstrate an effect of *MSSA* on late mortality. This may also be due to the virulence of this pathogen, causing patients to die within the first four weeks, rather than in the long-term. This is in accordance to a cohort study of patients with *S.aureus bloodstream infection* showing a high mortality of 46% within the first year [21].

#### Bacteremia

In a recent study Stangenberg et al. [16] showed a significant influence of bloodstream infection on mortality rates. We were also able to proof this effect as late mortality was found to be significantly influenced by the presence of bacteremia in our analysis. Detecting bacteremia as early as possible is crucial and could therefore reduce late mortality in VO.

#### Chronic kidney disease

Preoperative CKD was a predictive factor for both early and late mortality in VO patients. In a large-scale epidemiological analysis, Issa et al. were able to associate CKD with increased mortality [13]. Aagaard et al. also described a strong relationship between renal failure and mortality in their retrospective study [6]. They observed an increase in mortality risk of more than fivefold. Bains et al. explicitly investigated the influence of CKD on mortality after spinal surgery and attributed a special role to CKD [22]. Optimal treatment of CKD alongside with specific therapy for VO could thus have a significant impact on mortality rates. Factors leading to worsening renal function should be identified and, if possible, reduced or eliminated. These include optimal glycemic control in existing diabetes, blood pressure control and avoidance of nephrotoxic substances [23, 24]. Especially the latter plays an important role in the therapy of VO. Adequate pain therapy is one of the pillars in treatment. In the WHO staging scheme, non-opioid analgesics are provided as basic therapeutics. They can cause additional damage to the kidneys and should be used with caution. Substances used as part of anti-infective therapy should also be evaluated for their nephrotoxicity and, if necessary, administered in renal-adapted doses or alternative drugs should be used [23, 24].

### Strengths of the study

The current study includes a large number of patients and data from a prospective study with uniform 1-year follow-up, providing more detailed and robust information with strict inclusion criteria compared with case series or retrospective cohort studies. By differentiating between early and late mortality, our results give a more detailed view and provide important new clinically relevant insights into influencing factors and the course of this disease.

### Limitations

One limitation of the study is, that it is a single center study. Due to the retrospective design, there is a lack of data in single variables. Since missing data occur comparatively frequently in both groups and in particular the outcome variables mortality and recurrence are present in all patients, we assume that the data are missing at random and have no relevant influence on the analysis. Since our institution is a tertiary care centre, our cohort may have been influenced by referral bias; therefore, the results of the present study may not be generalizable. Because patients were often transferred from our maximum care centre to peripheral hospitals for continuation of anti-infective therapy, the duration of anti-infective therapy could not always be determined. Some parameters that would have been useful for this analysis, such as blood culture clearance or CKD stages, were not recorded in this database, but will be included in future prospective studies. Since the causes of death were not always recorded during follow-up, we unfortunately cannot distinguish whether patients died from reinfection, surgical complications, or general causes of death.

## Conclusion

Patients with chronic kidney disease or MSSA infection are at higher risk to die within the first 30 days of VO diagnosis. Patients who survive the first 30 days should be closely monitored and special attention should be paid on bacteremia and renal function during the first year, as these are the risk factors for late mortality, especially in elder patients. Consequently, special care should be taken when using potentially nephrotoxic substances (e.g. analgesics or antibiotics).

## Data Availability

The data that support the findings of this study are available on request from the corresponding author, NK. The data are not publicly available due to ethical restrictions.
